# Elective Node Irradiation With Integrated Boost to the Prostate Using Helical IMRT–Clinical Outcome of the Prospective PLATIN-1 Trial

**DOI:** 10.3389/fonc.2019.00751

**Published:** 2019-08-13

**Authors:** Stefan Alexander Koerber, Erik Winter, Sonja Katayama, Alla Slynko, Matthias Felix Haefner, Matthias Uhl, Florian Sterzing, Gregor Habl, Kai Schubert, Juergen Debus, Klaus Herfarth

**Affiliations:** ^1^Department of Radiation Oncology, Heidelberg University Hospital, Heidelberg, Germany; ^2^Heidelberg Institute of Radiation Oncology (HIRO), Heidelberg, Germany; ^3^National Center for Tumor Diseases, Heidelberg, Germany; ^4^Department of Statistics and Actuarial Science, University of Waterloo, Waterloo, ON, Canada; ^5^Radiation Oncology Unit, Strahlentherapie Süd, Kempten, Germany; ^6^Radiation Oncology Unit, Radiologie München, Munich, Germany; ^7^Heidelberg Ion-Therapy Center, Heidelberg, Germany; ^8^Clinical Cooperation Unit Radiation Oncology, German Cancer Research Center (DKFZ), Heidelberg, Germany; ^9^German Cancer Consortium (DKTK), Partner Site Heidelberg, Heidelberg, Germany

**Keywords:** prostate cancer, radiotherapy, pelvic nodes, IMRT, tomotherapy®, simultaneous integrated boost, elective node irradiation

## Abstract

**Introduction:** This prospective, non-randomized phase II trial aimed to investigate the role of additional irradiation of the pelvic nodes for patients with prostate cancer and a high risk for nodal metastases using helical intensity-modulated radiotherapy with daily image guidance (IMRT/IGRT).

**Methods and materials:** Between 2009 and 2012, 40 men with treatment-naïve prostate cancer and a risk of lymph node involvement of more than 20% were enrolled in the PLATIN-1 trial. All patients received definitive, helical IMRT of the pelvic nodes (total dose of 51.0 Gy) with a simultaneous integrated boost (SIB) to the prostate (total dose of 76.5 Gy) in 34 fractions. Antihormonal therapy (AHT) was administered for a minimum of 2 months before radiotherapy continuing for at least 24 months.

**Results:** After a median follow-up of 71 months (range: 5–95 months), pelvic irradiation was associated with a 5-year overall survival (OS) and biochemical progression-free survival (bPFS) of 94.3% and 83.6%, respectively. For our cohort, no grade 4 gastrointestinal (GI) and genitourinary (GU) toxicity was observed. Quality of life (QoL) assessed by EORTC QLQ-C30 questionnaire was comparable to EORTC reference values without significant changes.

**Conclusion:** The current trial demonstrates that elective IMRT/IGRT of the pelvic nodes with SIB to the prostate for patients with a high-risk of lymphatic spread is safe and shows an excellent clinical outcome without compromising the quality of life. The PLATIN-1 trial delivers eminent baseline data for future studies using modern irradiation techniques.

## Introduction

With an estimated incidence of 164,690 new prostate cancer cases in the United States in 2018, carcinoma of the prostate remains the most common malignancy in men (1). For intermediate and high-risk disease according to d'Amico criteria (2), surgery or radiotherapy are available curative, definitive treatment options. Although survival rates are much better compared to other malignant tumors, biochemical relapse occurred in a substantial proportion of patients. For dose-escalated irradiation, prostate-specific antigen (PSA) progression was reported in up to 35% of patients with intermediate or high-risk prostate cancer after 5 years (3). Many patients were diagnosed with lymph node metastases which are usually not included in the initial radiation field. Results from prostate-specific membrane antigen (PSMA) imaging showed positive, pelvic lymph nodes in up to 43.7% (4). Therefore, many studies focused on the role of whole pelvic radiotherapy (WPRT) including pelvic nodes. In 2003, Roach et al. observed a statistically significant improved progression-free survival for patients undergoing WPRT plus neoadjuvant and concurrent hormonal therapy (NCHT) in comparison with prostate-only irradiation (POI) (5). However, the benefit lost the level of significance with longer follow-up (6). This trial and several studies using more conventional radiation techniques reported on acute and/ or late gastrointestinal (GI) and genitourinary (GU) toxicities which occurred more frequently compared to POI (7–9). Moreover, there are some other trials questioning the clinical benefit of WPRT (10, 11).

By integration of modern radiation techniques like intensity-modulated radiotherapy (IMRT), a reduction of acute and late toxicities seems to be possible (12–14). The PLATIN-1 (Prostate and Lymph Node Irradiation with Integrated-Boost-IMRT after neoadjuvant hormonal therapy [NHT]) trial evaluates the role of modern IMRT/ image-guided radiotherapy (IGRT) technique for treatment-naïve prostate cancer patients undergoing optimized WPRT. By using a moderately hypofractionated, simultaneous integrated boost (SIB) to the prostate, the current study also analyzes the influence of moderate hypofractionation on biological effectiveness in a definitive treatment setting after NHT (15). The present article reports on late toxicity and clinical outcome of this cohort.

## Materials and Methods

### Study Participants and Procedures

The present study was approved by the local ethics review board (S-034/2009). In total, 40 men with treatment-naïve and histologically proven prostate cancer were prospectively enrolled in the PLATIN-1 trial between May 2009 and December 2012. All patients had no suspicious lymph node in pelvic computed tomography (CT) or magnetic resonance imaging (MRI) and an estimated risk of pelvic lymph node involvement exceeding 20% according to the Roach formula {2/3 PSA + [(GS−6) × 10]}(16). Antihormonal therapy (AHT) was authorized for all patients and consisted of a minimum of 2 months neoadjuvant treatment and the advice of continuation for at least 24 months after irradiation if tolerated. AHT included luteinizing hormone-releasing hormone (LHRH) agonists or antiandrogen medication.

Treatment planning and radiation were performed as described previously (15). In summary, patients were irradiated once daily and five fractions a week. The prescribed dose of 95% of the planning target volume of the pelvic lymph nodes (PTV-L) was 51.0 Gray (Gy) with a single dose of 1.5 Gy. A simultaneously integrated boost (SIB) of 76.5 Gy was prescribed to 95% of the PTV prostate (PTV-P) with a single dose of 2.25 Gy. Irradiation was performed with helical IMRT/ IGRT using a Tomotherapy® system (Accuray, USA).

### Follow-Up and Assessment of Toxicity and Quality of Life (QoL)

Before irradiation, during treatment (weekly) and at the end of the treatment prostate-specific symptoms and treatment toxicity were graded according to the National Cancer Institute Common Terminology Criteria for Adverse Effects (NCI CTCAE) version 3.0. Assessment of QoL using the European Organization for Research and Treatment of Cancer (EORTC) Quality of Life Questionnaire-Core 30 (QLQ-C30) was first performed before treatment. The PSA level was assessed every 3 months. The follow-up schedule included visits at 2.5, 6, 12, 18, and 24 months including toxicity records and QoL records (performed only at 6, 12, and 24 months). Patients were regularly followed thereafter based on local standard operating procedures. This included measurement of PSA levels and toxicity assessment. The median follow-up was 71 months.

### Statistical Analysis

The primary objective was the examination of biochemical progression-free survival (bPFS), clinical relapse-free survival (cRFS) and overall survival (OS) for patients suffering from non-metastatic prostate cancer undergoing both IGRT/IMRT and AHT. Furthermore, the secondary objectives were to examine late toxicity and prostate specific symptoms. Biochemical failure was defined according to the Phoenix criteria (17), clinical failure was defined as the existence of local recurrence or metastases detected by CT including PET-CT, MRI or bone scan, which were performed after clinical evidence based on symptoms. The Kaplan-Meier method was used for calculating bPFS, cRFS, and OS.

All statistical analyses were performed by using SPSS v.25.0 and a *P*-Value of < 0.05 was defined as significant.

## Results

### Patients

Due to an increase of PSA levels during NHT, two patients were excluded from the study before radiotherapy. The patient's characteristics of the remaining 38 were previously described by Habl et al. (15). Median age was 70.5 years with a range of 51–75 years. According to the Roach formula, a risk of LNI of more than 40% was calculated for 6 patients (15.8%) of the cohort while 32 patients (84.2%) had a risk of 20–40%. Twenty-seven patients (71.1%) received LHRH agonists, seven patients (18.4%) antiandrogen therapy (bicalutamide) and four patients (10.5%) both (complete androgen deprivation). Only 8 patients (21.1%) received AHT for the required period of 24 to 36 months. Twelve patients (31.6%) stopped AHT within 6 to 24 months of follow-up, 13 patients (34.2%) after a maximum period of 5 months (including NHT) due to intolerance or side effects. AHT was continued for five patients (13.2%) until the current evaluation (Table 1). For all patients, irradiation was performed as specified in the protocol.

**Table 1:** Patient's characteristics.

**Characteristics**	**Number of patients**
Number of patients	38
Age [years], median (range)	70.5 (51–75)
**T-Stage, *n* (%)**	
T1	21 (55.2%)
T2	8 (21.1 %)
T3	8 (21.1%)
T4	1 (2.6%)
**Gleason score, *n* (%)**	
≤6	0 (0.0%)
7	18 (47.4%)
≥8	20 (53.6%)
iPSA [ng/ml], median (range)	17.5 (0.5–120.0)
**Risk-group according to d'Amico, *n* (%)**	
Low	0 (0.0%)
Intermediate	3 (7.9%)
High	35 (92.1%)
**Risk of LNI according to Roach formula *n* (%)**	
>20–40%	32 (84.2%)
> 40 %	6 (15.8%)
**AHT**	
<24 months	25 (65.8%)
24–36 months	8 (21.1%)
>36 months	5 (13.2 %)

*LNI, lymph node involvement; AHT, antihormonal therapy*.

### Clinical Outcome

**Figure 1:**
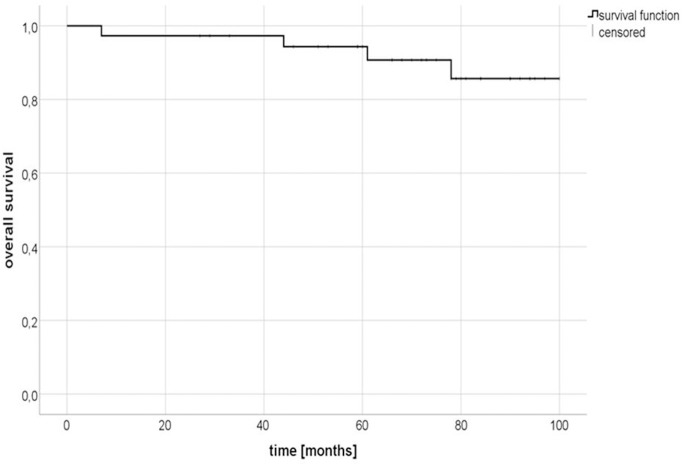
Kaplan-Meier estimates of overall survival (OS).

**Figure 2:**
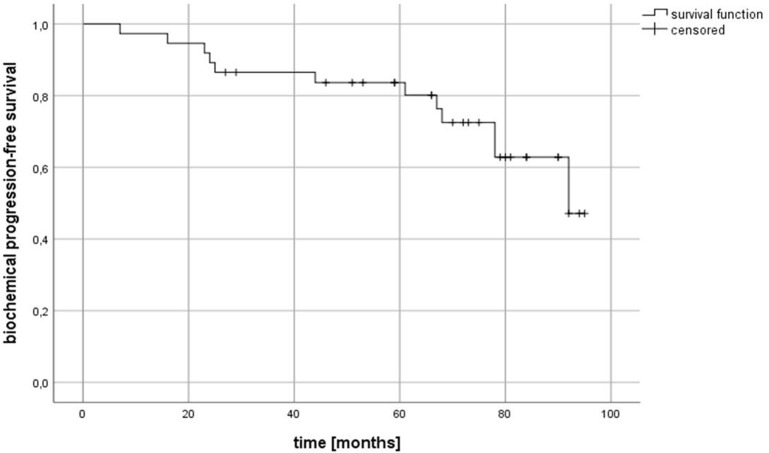
Kaplan-Meier estimates of biochemical progression-free survival (bPFS).

After a median follow-up of 71 months (range: 5–95 months), 34 out of 38 patients (89.5%) were still alive. One patient died almost 7 months after irradiation due to a newly diagnosed, metastasized esophageal cancer. One patient died after 61 months due to cardiac disease, another after 44 months due to acute myeloic leukemia. For one patient, the reason for death is unknown. The 2-year and 5-year overall survival (OS) rates were 97.3% (95% confidence interval [CI] 96.4–98.2%) and 94.3% (95% CI 93.1-95.6%), respectively (Figure 1). In 21.5% (8 patients) of the cohort, a biochemical relapse occurred. For four patients with PSA relapse, further imaging with MRI, CT and/ or bone scan was performed. One patient was diagnosed with local recurrence, two patients with bone metastases. No nodal relapse within the pelvis occurred. A biochemical progression-free survival (bPFS) of 89.2% (95% CI 87.6–90.8%) and 83.6% (95% CI 81.6–85.6%) was observed at 2 and 5 years, respectively (Figure 2).

### Late Toxicity

At the time of last follow-up, toxicity data were available for 29 patients. We observed no grade 3 and 4 late toxicity with regard to gastrointestinal (GI) side effects. Two men (5.3%) reported on grade 1 enteritis, one patient (2.6%) on grade 2 enteritis with pain and moderate bleedings. No proctitis or diarrhea occurred in our cohort at the time of follow-up.

For patients undergoing helical IMRT, there was no grade 4 genitourinary (GU) toxicity. The cumulative incidence of grade 3 urinary side effects was 2.6% including one patient with stress incontinence. Urge incontinence occurred for 9 patients (23.7%; grade 1) and 3 patients (7.9%; grade 2), respectively. Only one patient (2.6%) reported on a light cystitis (grade 1). Without current AHT, five patients (13.1%) reported on grade 2/3 erectile dysfunction, while grade 2/3 loss of libido was found for 16 patients (42.1%). Two patients (5.3%) were identified with grade 1 edema and three patients (7.9%) with grade 2 edema at the time of follow-up. No grade 3 or 4 edema was observed for the entire cohort (Table 2).

**Table 2:** Late toxicity (median follow up: 71 months; *n* = 38).

**Characteristics**	**Grade 0**	**Grade 1**	**Grade 2**	**Grade 3**	**Unknown**
**Gastrointestinal (GI) side effects**					
Enteritis	26 (68.4%)	2 (5.3%)	1 (2.6%)	0 (0.0%)	9 (23.7%)
Proctitis	29 (76.3%)	0 (0.0%)	0 (0.0%)	0 (0.0%)	9 (23.7%)
Diarrhea	29 (76.3%)	0 (0.0%)	0 (0.0%)	0 (0.0%)	9 (23.7%)
**Genitourinary (GU) side effects**					
Cystitis	28 (73.7%)	1 (2.6%)	0 (0.0%)	0 (0.0%)	9 (23.7%)
Urge incontinence	17 (44.7%)	9 (23.7%)	3 (7.9%)	0 (0.0%)	9 (23.7%)
Stress incontinence	22 (57.9%)	0 (0.0%)	6 (18.4%)	1 (2.6%)	9 (23.7%)
Dysuria	16 (42.1%)	1 (2.6%)	1 (2.6%)	0 (0.0%)	20 (52.6%)
**Erectile dysfunction**					
> Without current AHT	2 (5.3%)	4 (10.5%)	1 (2.6%)	4 (10.5%)	24 (63.2%)
> With current AHT	0 (0.0%)	0 (0.0%)	0 (0.0%)	1 (2.6%)	2 (5.3%)
**Loss of libido**					
> Without current AHT	1 (2.6%)	7 (18.4%)	7 (18.4%)	9 (23.7%)	11 (28.9%)
> With current AHT	0 (0.0%)	0 (0.0%)	0 (0.0%)	3 (7.9%)	0 (0.0%)
Edema	22 (57.9%)	2 (5.3%)	3 (7.9%)	0 (0.0%)	11 (28.9%)

*AHT, antihormonal therapy*.

## Discussion

After a median follow-up of 71 months, IMRT/ IGRT of the prostate and pelvic nodes continued to be well-tolerated without excessive side effects. For our cohort of 38 men treated in the present study, no severe (grade 3/4) GI toxicity occurred. The Genitourinary Study Group (GETUG)-1 trial – one of the largest prospective studies investigating the role of pelvic node irradiation–reported on a grade 3/4 toxicity rate for the digestive tract of 10.7% after a median follow-up of 42.1 months. In this trial, irradiation was performed with a four-field box to a total dose of 46 Gy to the pelvis and a maximum of 70 Gy to the prostate (10). Although total dose to the prostate was lower compared to our PLATIN-1 trial according to former guidelines, the reduced number of side effects in the present study can be explained by the use of modern treatment techniques like IMRT in combination with daily imaging (IGRT). This is in accordance with other studies using IMRT: Pervez et al. observed no grade 3/4 late GI toxicity in a group of 60 patients undergoing irradiation of the pelvic nodes and prostate (total dose: 45/68 Gy) in 25 fractions at 5 years follow-up timepoint (18). Similar results were described for GU side effects, however, a direct comparison is difficult due to a lack of detailed data in the majority of other reports and a limited number of feedbacks in our cohort. In the present study, 68.9% of the patients were unwilling or unable to provide any information about their erectile function. Nevertheless, in addition to the reported grade 2/3 erectile dysfunction rate of 14.3% for the current study, a high number of genital constraints might automatically result from AHT and the increasing age of the patients. In the Prostate Testing for Cancer and Treatment (ProtecT) trial, a group of 1,643 men with a median age of 62 years was included. At 72-months follow up, erection not firm enough for intercourse was found for 73% in the radiotherapy compared to 70% in the active surveillance (AS) group (19). Even watchful waiting caused similar limitations in 80% of men in the Scandinavian Prostate Cancer Group-4 at 144-months follow-up, although the median age of this cohort was also younger (64 years) than that of the present PLATIN-1 trial (70.5 years) (20). Age might the relatively high rate of incontinence in our cohort. In total, 21% reported on grade 2/3 stress incontinence while urge incontinence was observed for 7.9% at the time of follow-up. In a phase 1/2 dose-escalation study from UK, the 2-year cumulative rates of grade 2+/grade 3+ bladder toxicity were 4.2%/ 4.2% (cohort 1), respectively. This study investigated the role of IMRT to the prostate (total dose of 70 to 74 Gy) and pelvic lymph nodes (total dose for cohort 1: 50 Gy) including 25 patients with prostate cancer (21). However, our cohort also showed high rates of incontinence before irradiation. Almost 16 % of men included in the PLATIN-1 trial complained about grade 1/2 incontinence at baseline. Overall quality of life assessed by the EORTC QLQ-C30 questionnaire remained largely stable at 71-months follow-up. Global health score was 68.1, which is in accordance with EORTC reference values of prostate cancer patients. Compared to month 24, there was a significant improvement of global health status (15). One explanation might be that also protracted, radiotherapy-related symptoms disappeared and AHT was finished for almost all patients. Our observations are at least comparable to a recent report published by Lips et al. comparing QoL in patients with locally advanced prostate cancer after 76 Gy IMRT vs. 70 Gy conformal radiotherapy. The authors concluded that dose-escalated IMRT/ IGRT can be performed without deterioration in QoL (22). The expansion of the target volume by adding pelvic lymph nodes also seems to cause no substantial change, if modern radiation technique is used. At least in our cohort of 38 men undergoing helical IMRT, no significant variations for QoL scores were observed compared to reference values.

However, one crucial question remains: Is there an oncological benefit for pelvic node irradiation in non-metastatic patients with prostate cancer? While several retrospective and small prospective studies report on promising results (9, 18, 21, 23–25), two randomized phase III trials failed to show an improved survival for patients undergoing pelvic irradiation (Table 3): The last update of the GETUG-01 randomized study evaluating 446 men with prostate carcinoma summarized, that pelvic nodes irradiation was not able to improve event-free survival (EFS) or OS after a median follow-up of 11.4 years (11). For the RTOG 9413 cohort including 1,322 patients, an improved PFS was observed for NHT plus WPRT compared with NHT plus prostate-only radiotherapy (PORT) and WPRT and adjuvant hormonal therapy after a median follow-up of 8.8 years. Nevertheless, WPRT did not show an improvement in OS compared to PORT while leading to an increased risk of grade 3 or worse GI toxicity with the use of conventional four-field technique (6). In the present trial, IMRT/ IGRT of the pelvic lymph nodes with a simultaneous integrated boost to the prostate achieved no nodal relapse and excellent 5-year bPFS and OS of 83.6% and 94.3% considering the high rate of high-risk patients and short-term (<24 months) AHT. Although the PLATIN-1 trial was a prospective trial using modern radiation technique, the current study was not powered to provide sufficient data regarding oncological outcome. Due to the small number of patients, the non-randomized setting and a certain number of men with only short-term AHT–major limitations of our study—there is still a lack of evidence regarding prophylactic irradiation of pelvic nodes for patients with prostate cancer. Both, the GETUG-01 and the RTOG 9413 were not able to show a general benefit for WPRT, however, from today's view several parameters could limit the results of them: Besides broad inclusion criteria (GETUG-01) and a low total dose according to former guidelines, the four-field technique without image guidance could have resulted in insufficient doses within some areas like the presacral or external iliac nodes. Therefore, the present PLATIN-1 trial formed a solid basis for ongoing trials using modern photon or proton irradiation and was an important contribution to evaluate prophylactic pelvic node irradiation using IMRT/ IGRT, but more evidence is needed about whether or not an expanded target volume is beneficial to men with non-metastatic, high-risk prostate cancer. With the end of recruitment for one upcoming study, the RTOG 0924 trial, expected by late summer 2019 (6), further information should be available.

**Table 3:** Overview of prospective trials evaluating the role of WPRT.

**References**	**Trial design/number of patients (*n*)**	**Radiation technique**	**Total/single dose pelvic nodes**	**Total/ single dose prostate**	**AHT**	**Follow-up**	**Results**
Adkinson et al. (23)	Prospective, non-randomized phase I trial; *n* = 53	Helical IMRT or step-and-shoot IMRT	56.0/2.0 Gy	70/2.5Gy	88.7% for 6–28 months	25.4 months	Preliminary biochemical control of 81.2% at 3 years; No grade 3+ late GI toxicity, one grade 3 GU toxicity
Di Muzio et al. (26)	Single-center, prospective, non-randomized phase I-II trial; *n* = 211	Helical IMRT	51.8/ 1.85 Gy (for intermediate- and high-risk)	71.4/2.55 Gy or 74.2/ 2.65 Gy	Intermediate risk: 12 months; high-risk 36 months	5 years	5-year bRFS 93.7%, 5-year OS 88.6%; Late grade 3+ toxicity of 5.9% (GU) and 6.3% (GI)
Magli et al. (24)	Single-center, prospective, non-randomized phase II trial; *n* = 41	Step-and-shoot IMRT	50.0/ 2.0 Gy	67.5/ 2.7 Gy	12–24 months	65.4 months	5-year bRFS 95.1%; No grade 3+ late toxicity
Pervez et al. (18, 27)	Single-center, prospective, non-randomized phase II trial; *n* = 60	Helical IMRT	45.0/ 1.8 Gy	68.0/ 2.7 gy	24–36 months (NHT up to 6 months)	63 months	5-years OS 86.7%; 5-year freedom from biochemical failure 91.7%; No grade 3+ GI toxicity; grade 3 GU toxicity 2.4%
Pommier et al. (GETUG-01) (11)	Multicenter, prospective randomized trial; *n* = 446	Conventional four-field technique	46.0/2.0 Gy or 46.8/1.8 Gy or 45.0/ 2.25Gy^*^	66.0-70.0/2.0 Gy or 68.4–72.0/1.8 Gy or 65.25–69.75/2.25 Gy^*^	High-risk: NHT for 4-8 months and concomitant (about 60% in each arm)	11.4 years	10-year EFS 57.6% (WPRT) vs. 55.6% (PORT); 10-year OS 74.9% (WPRT) vs. 73.6% (PORT);
Roach et al. (RTOG 9413) (6)	Multicenter, prospective randomized trial (2 × 2 factorial design); *n* = 1,323	Conventional four-field technique	50.4/ 1.8 Gy	70.2/1.8Gy	NHT: 2 months and during RT adHT: start with RT	8.8 years	10y-PFS 28.4% (NHT+WPRT)/ 23.5% (NHT+PORT)/ 19.4% (WPRT+adHT)/30.2% (PORT+adHT); No OS difference (346 patients alive); late grade 3+ GI toxicity of 7% for NHT+WPRT

* *only 4 fractions/ week 3D-CRT, 3D-conformal radiotherapy; adHT, adjuvant hormonal therapy; AHT, antihormonal therapy; bRFS, biochemical relapse-free survival; BDFS, biochemical disease-free survival; EFS, event-free survival; NHT, neoadjuvant hormonal therapy; GI, gastrointestinal; GU, genitourinary; IMRT, intensity modulated radiation therapy; OS, overall survival; PORT, prostate-only radiotherapy; WPRT, whole pelvis radiotherapy*.

In summary, the present PLATIN-1 trial confirms that helical IMRT of the pelvic nodes with a simultaneous integrated boost to the prostate can be performed without severe toxicity and significant deterioration in QoL. Even when our trial achieved excellent oncological outcome, there is still a need for further randomized studies evaluating the role of prophylactic, pelvic irradiation for patients with prostate carcinoma and a high risk for LNI.

## Data Availability

All datasets generated for this study are included in the manuscript/supplementary files.

## Author Contributions

SAK and EW were the main investigators who collected and evaluated the clinical information. AS conducted the statistical analysis and critically evaluated the paper. SK, MH, MU, FS, and GH assisted the main author in patient enrollment, data collection and revising the final draft. The paper was wrote by SAK and critically evaluated by KS, KH, and JD. KH and JD designed and supervised the prospective trial. All authors read and approved the final version of the paper.

### Conflict of Interest Statement

The authors declare that the research was conducted in the absence of any commercial or financial relationships that could be construed as a potential conflict of interest.
